# Delivery of HIV care during the 2007 post-election crisis in Kenya: a case study analyzing the response of the Academic Model Providing Access to Healthcare (AMPATH) program

**DOI:** 10.1186/1752-1505-7-25

**Published:** 2013-12-01

**Authors:** Suzanne Goodrich, Samson Ndege, Sylvester Kimaiyo, Hosea Some, Juddy Wachira, Paula Braitstein, John E Sidle, Jackline Sitienei, Regina Owino, Cleophas Chesoli, Catherine Gichunge, Fanice Komen, Claris Ojwang, Edwin Sang, Abraham Siika, Kara Wools-Kaloustian

**Affiliations:** 1 Department of Medicine, Indiana University School of Medicine, Indianapolis, USA; 2 Academic Model Providing Access to Healthcare (AMPATH), Eldoret, Kenya; 3 Department of Epidemiology, School of Public Health, College of Health Sciences, Moi University, Eldoret, Kenya; 4 Department of Medicine, School of Medicine, College of Health Sciences, Moi University, Eldoret, Kenya; 5 Divison of Global Health, Dalla Lana School of Public Health, University of Toronto, Toronto, Canada; 6 Regenstrief Institute, Indianapolis, USA; 7 Department of Health Policy and Management, School of Public Health, College of Health Sciences, Moi University, Eldoret, Kenya; 8 School of Public Health, Griffith Health Institute, Griffith University, Southport, Queensland, Australia; 9 Moi Teaching and Referral Hospital, Eldoret, Kenya

**Keywords:** HIV/AIDS, Antiretroviral therapy, Kenya, Violence, Crisis

## Abstract

**Background:**

Widespread violence followed the 2007 presidential elections in Kenya resulting in the deaths of a reported 1,133 people and the displacement of approximately 660,000 others. At the time of the crisis the United States Agency for International Development-Academic Model Providing Access to Healthcare (USAID-AMPATH) Partnership was operating 17 primary HIV clinics in western Kenya and treating 59,437 HIV positive patients (23,437 on antiretroviral therapy (ART)).

**Methods:**

This case study examines AMPATH’s provision of care and maintenance of patients on ART throughout the period of disruption. This was accomplished by implementing immediate interventions including rapid information dissemination through the media, emergency hotlines and community liaisons; organization of a Crisis Response leadership team; the prompt assembly of multidisciplinary teams to address patient care, including psychological support staff (in clinics and in camps for internally displaced persons (IDP)); and the use of the AMPATH Medical Records System to identify patients on ART who had missed clinic appointments.

**Results:**

These interventions resulted in the opening of all AMPATH clinics within five days of their scheduled post-holiday opening dates, 23,949 patient visits in January 2008 (23,259 previously scheduled), uninterrupted availability of antiretrovirals at all clinics, treatment of 1,420 HIV patients in IDP camps, distribution of basic provisions, mobilization of outreach services to locate missing AMPATH patients and delivery of psychosocial support to 300 staff members and 632 patients in IDP camps.

**Conclusion:**

Key lessons learned in maintaining the delivery of HIV care in a crisis situation include the importance of advance planning to develop programs that can function during a crisis, an emphasis on a rapid programmatic response, the ability of clinics to function autonomously, patient knowledge of their disease, the use of community and patient networks, addressing staff needs and developing effective patient tracking systems.

## Background

**Figure 1:**
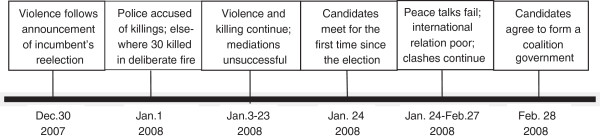
Timeline of 2007 post –election violence and negotiations.

Kenya has a recent history of ethnic violence, with conflicts arising following the national elections in 1992 and 1997. In 2007, the presidential election was held on December 27. Three days later the tallying of votes was completed and the incumbent candidate was announced the winner. These results were contested by the challenger’s party with allegations of election malpractice. Widespread ethnically-related violence broke out on December 30, 2007 and lasted through March 2008 (Figure 1). During this period 1,133 individuals were killed, 78,000 homes were destroyed or looted and 660,000 people were displaced from their homes [1]. An estimated 350,000 of those displaced were forced to seek refuge in Internally Displaced Persons (IDP) camps [1-3]. Due to disruptions in transport, personal displacement, disruption of medical supply chains and safety issues, many clinics experienced operational difficulties and patients had trouble accessing care. As such, in the geographic areas most heavily affected by violence potential disruptions in patient’s antiretroviral therapy (ART) became a significant concern [4-7].

Successful delivery of ART in Africa is possible in adults and children as evidenced by good clinical and immunological responses [8,9]. However, disruptions in ART can lead to antiretroviral resistance resulting in virologic failure and subsequent immunologic and clinical failure [6-14]. To minimize these negative outcomes, continued ART delivery is recommended as a part of HIV services in emergency settings [15,16] and guidelines have been published [17,18]. However, reports detailing recommendations for implementation and best practices to maintain delivery of HIV care in the face of societal disruptions caused by war, systemic violence or natural disasters are limited to the experience of a small number of groups [19-21].

The USAID-Academic Model Providing Access to Healthcare (AMPATH) Partnership has operated an HIV care and treatment program in western Kenya since November 2001. Many clinic sites are located in geographic areas where the post-election violence was concentrated. The purpose of this paper is to document the AMPATH’s response to the crisis and the adaptations that the program used to provide continuity of care in the face of social instability, as well as to outline lessons learned and to provide recommendations for other ART programs that may face similar challenges.

## Methods

### Structure of AMPATH

#### Program history

In 2001, Indiana University School of Medicine (IUSM) (Indianapolis, Indiana) and Moi University Faculty of Health Sciences (Eldoret, Kenya) in collaboration with the Moi Teaching and Referral Hospital, created AMPATH in response to the rapidly increasing HIV patient population being cared for at the Referral Hospital [22]. The goal of this program was to provide HIV care in both urban and rural settings in western Kenya. In 2004, with funding from the President’s Emergency Plan for AIDS Relief (PEPFAR) and the United States Agency for International Development (USAID), the USAID- AMPATH Partnership (subsequently identified as AMPATH) was created.

#### AMPATH Clinics

**Figure 2:**
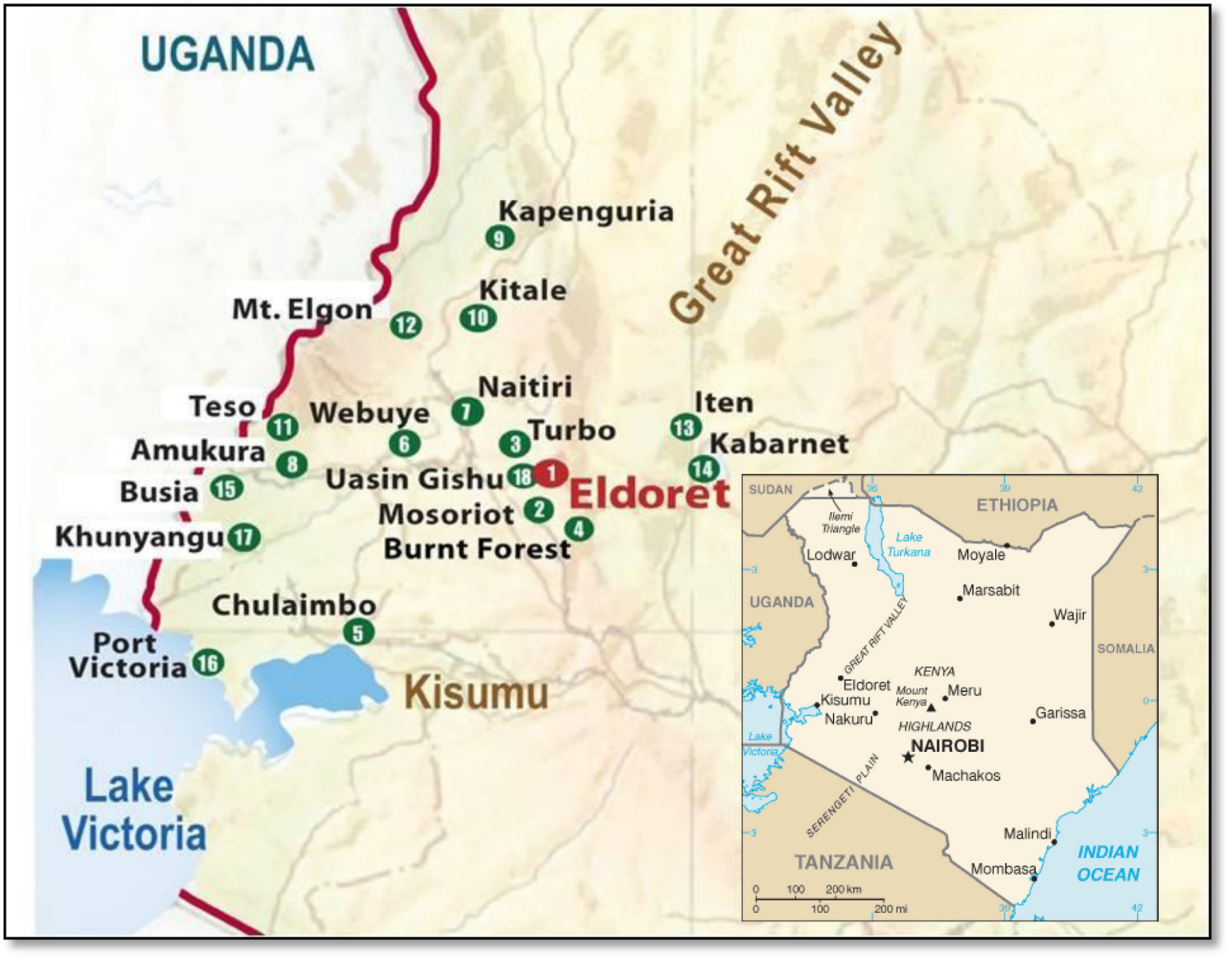
AMPATH clinics as of January 1, 2008.

In December 2007, at the time of the post-election violence, AMPATH was treating 59,437 HIV infected patients (39% on ART) at 17 Ministry of Health clinics in western Kenya (Figure 2). The Eldoret clinic acted as the program hub, receiving daily deliveries of patient data and laboratory samples from outlying clinics. Following patient visit data entry and laboratory specimen processing in Eldoret, forms and test results were returned to outlying clinics. Distribution of ART, other medications and food to the outlying clinics also originated from the main pharmacy and stocks in Eldoret. The Eldoret clinic itself was composed of four independently functioning modules designated as 1a-1d (3 adult clinics and 1 pediatric clinic housed in a single building) for reporting purposes. The other clinic sites active at the time of the crisis were located in Mosoriot, Turbo, Burnt Forest, Chulaimbo, Webuye, Naitiri, Amukura, Kapenguria, Kitale, Teso, Mt. Elgon, Iten, Kabarnet, Busia, Port Victoria and Khunyangu (Figure 2). Uasin Gishu clinic opened for enrollment just prior to the election crisis and was not included in this report. All of these clinics are located in western Kenya within four hours by road from the main clinic in Eldoret. Some clinics operate satellite clinics, small clinics with limited services open 1–4 days per month and staffed by clinicians from the main clinic. The clinic in Burnt Forest was the most dramatically affected, but the clinics in Eldoret, Mosoriot, and Turbo were also located in the areas of great political instability and violence.

#### Burnt Forest clinic

We will describe the response of the AMPATH clinics as a whole, but because Burnt Forest was the most profoundly impacted clinic within the system it will serve as an embedded case study throughout the remainder of this report. The Burnt Forest Health Center HIV Clinic was the third rural clinic, founded in 2003. It is located approximately 30 minutes by road from the main clinic in Eldoret, and at the time of the election the clinic cared for 1,747 HIV infected individuals, 57% of whom were receiving ART. This community has experienced violence during prior elections, including the 2007 election, largely due to the mixed ethnic make-up of the population including tribes supporting the prior president and those supporting the opposition party. An estimated 1,320 homes (housing approximately 5,000 people) as well as numerous farms and businesses were destroyed during the early days of the violence [2]. As a result supporters of the prior president relocated to the town center and opposition supporters moved to farms on the outside of town. Thus travel to clinic was complicated by concerns about having to cross areas held by ethnic groups supporting the rival party.

#### Standard of care

At the time of the 2007 election the majority of patients receiving ART were scheduled for monthly clinic visits which required, at minimum, contact with a nurse, a clinician, and a pharmacy technician (or nurse). High risk patients (CD4 counts ≤ 100 cells/μl at ART initiation or history of poor compliance) were seen more frequently (every 1–2 weeks). At each routine clinic visit patients were provided with a one month supply of antiretrovirals and prophylactic medications free of charge. On some occasions patients received more than a month’s supply of drugs secondary to special circumstances (planned travel out of the catchment area, distance from home to clinic, scheduled holidays, etc.). Laboratory testing was undertaken per protocol and for clinical indications.

Patients were evaluated for food insecurity at enrollment and upon referral by a clinician. Supplemental food, procured through the program’s farm initiative and the World Food Program (WFP), was provided for the families of all food-insecure patients.

A member of the clinic’s Outreach Program interviewed all patients enrolling into the program and documented their contact information on a locator form that included the patient’s cell phone number and a map to their home with key landmarks documented (proximity to schools, churches, shops, etc.). This information was used to locate patients who did not return for their clinic appointment within a designated time frame.

#### AMPATH Medical records system (AMRS)

The clinics utilize the AMPATH medical record system (AMRS), an electronic medical records system designed to collect and manage patient level data, rather than the aggregate data collected by Kenya’s National Health Information System. It is used for clinical care, program reporting and research purposes [23,24]. Paper-based initial and follow-up forms (adult and pediatric) are completed by the clinician during a clinic visit. These standardized forms capture demographics, clinical, laboratory and pharmacologic information. At the time of the election all completed forms were being transported to Eldoret for data entry and once entered were subsequently returned to the clinics. The AMRS database was not directly accessible at the outlying clinics.

### Structural response

Although the potential for instability in areas serviced by AMPATH clinics could be anticipated by the history of election violence, AMPATH clinics had not previously operated during such outbreaks. As such, planned clinic closures and distribution of additional ART to patients were in place to coincide with the Christmas and New Year holidays as well as the elections, but no formal structural response plan had been developed. In immediate response to the disruption caused by the post-election violence, AMPATH’s administration performed a rapid assessment of its clinics and the needs of its HIV-infected patients on January 6, 2008. This resulted in the development of a multifaceted disaster response plan consisting of five primary components: 1) *Mobile Clinics and Outreach*: this arm was designed to provide clinical services at the Internally Displaced Persons (IDP) camps, established a system to refer patients to the AMPATH clinic operating closest to their current location, and identify and track patients who had missed clinic appointments. 2) *Administration and Logistics arm*: This arm provided physical and logistical assistance to the teams visiting the IDP camps. In conjunction with the IU-Kenya program administrative office (Indiana), this team was also responsible for the management of emergency recovery funds and procurement of relief supplies. 3) *Media and Information*: This arm designed and facilitated electronic and print media announcements in national and vernacular languages. 4) *Patient and Staff Humanitarian Assistance*: This arm was responsible for assessing and responding to the socio-economic needs of the distressed patients and staff. 5) *Monitoring and Evaluation arm*: This arm was responsible for tracking and reporting patient care in the IDP camps, identifying gaps in care within the IDP camps and developing interventions to address these gaps. As part of this planning AMPATH decided not to engage in widespread provision of basic relief services (i.e. hygiene, sanitation, water, food, security, immunizations), as many international organizations with expertise in this area were already responding to the crisis. In order to implement the response plan, AMPATH staff divided into five teams with each team assigned to a response arm. The leadership worked to coordinate efforts with other governmental and non-governmental agencies.

### Data collection and management

#### IDP Camps

A “Field Encounter Form – Internally Displaced AMPATH Patients” was developed and used primarily for patient encounters in the IDP camps, but also for those patients displaced to other locations or unable to travel to clinic due to safety concerns. In addition to the demographic, clinical and pharmacologic information recorded on the routine clinical forms, this form collected information on patient affiliation (AMPATH versus non-AMPATH), IDP camp location if applicable, status of the patient’s home (standing versus destroyed) and whether antiretroviral treatment had been interrupted due to the election crisis. The standard AMPATH Prescription Form was used to dispense medications.

#### AMPATH Clinics

To assess the impact of the post-election crisis on clinic function, all clinic sites received an “AMPATH Post-Crisis Clinic Evaluation”. This form was designed to collect data on the overall function of the clinic including information on the date of scheduled clinic opening (post-Christmas and New Year’s holidays), date of actual clinic opening, number of staff and clinical officers available, ancillary services available, methods used for patient data collection, availability of antiretrovirals, and the number of patients seen by the clinics at one, two and three weeks post-election as well as at one and two months. Clinic documentation of patient visits was requested since some clinics were unable to complete routine follow-up forms either due to patient volume or supply issues. As a consequence not all visits were captured in the AMRS during this period.

#### Emergency hotline logs

Emergency Hotline calls were answered by a Clinical Officer (CO) or nurse. For each call they logged the caller’s name, phone number, area calling from, problem, advice and their signature.

#### Burnt Forest clinic patient logs

Utilizing the AMRS, a list of all AMPATH patients enrolled at Burnt Forest clinic on ARVs was generated. The list included patient name, age, AMPATH ID number, village/location, last clinic visit date, expected return date, ART status (on ART or not on ART) and the patient’s phone number if known.

### Data analysis

Data were extracted from the AMRS for the 6 months prior to the post election violence in order to determine the mean number of clinic visits per month at each clinic. A percentage of the mean patient volume per clinic was calculated by taking the reported clinic attendance weekly during each interval surveyed and dividing by the mean number of clinic visits per week over the prior 6 months. Simple proportions were calculated for the number of scheduled staff present in clinic during each surveyed time period.

## Results

### Information distribution

#### Media messages

On January 8, 2008 public service messages were aired on five local radio stations and published in a daily national newspaper. Although AMPATH authored and distributed the initial messages, this task was subsequently taken over and continued by the Kenyan MOH. The messages included: all AMPATH clinics are open for care and ARV distribution; the importance of not missing antiretroviral doses; the ability of any HIV infected patient to access antiretrovirals at any AMPATH or Kenyan MOH clinic by showing their empty pill bottles or clinic appointment card; emergency hotline numbers for care questions and directions for correctly stopping medications if unable to access care. For those patients utilizing infant formula, information was given on obtaining formula from any AMPATH clinic, hotline numbers for those unable to access a clinic and caution against mixing breastfeeding with other sources of milk.

Hotline numbers were published as part of the public service information provided by the program. Patients could text or “flash” their phone number to the hotline numbers and receive a return phone call from a CO or nurse. The hotline staff logged 212 calls in the three weeks following the number’s dissemination. Calls were received from both AMPATH and non-AMPATH patients and advice was provided on a number of topics, with the most frequent being how to: obtain antiretroviral refills, access care at a local hospital, access AMPATH care and obtain HIV-testing. For those callers seeking assistance in finding a clinic near their current location, hotline personnel had a list of 358 hospitals and clinics located throughout Kenya to which callers could be referred.

### Clinic operations

#### Clinic Re-openings and Staffing

**Figure 3:**
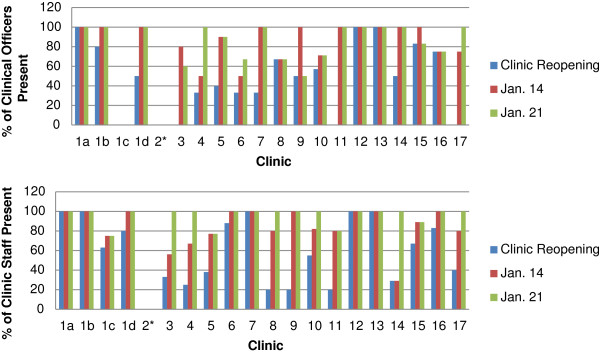
Availability of clinical officers and clinic staff at clinic reopenings and in the following weeks.

The majority of the twenty clinics (75%) reopened on their scheduled dates following the holidays (Figure 3). Of the five clinics that did not open as scheduled, four were in areas highly affected by violence, including Burnt Forest. Ultimately, all of these clinics were open within five days of their planned reopening (two of the late opening clinics were modules located in Eldoret and were covered by the other modules at the site). At the time of reopening, only five clinics (25%) had the full number of expected non-clinician staff (includes nurses, pharmacists, nutritionists and social workers) and three (17%) had the full number of expected COs. However, by the end of the first week, no clinic was operating with less than 50% of their staff, nine clinics were operating with full staff and ten clinics with all clinicians. By the end of the third week 89% of clinics had returned to full staff and 68% of clinics had their full complement of COs. All clinics were considered to be staffed sufficiently to provide services at pre-violence levels without compromise of service, thus no subsequent surveys were conducted. In order to compensate for the decreased number of COs and staff, patient encounter were brief and in some cases documentation was minimized.

#### Burnt Forest clinic staffing

At the Burnt Forest clinic only two staff members (a CO and a nurse) of the expected seven were able to return to the clinic on time. In the three days following the outbreak of violence, these staff members treated any patient presenting to the clinic regardless of their HIV status. The majority of patients presented with injuries related to the violence, such as burns, arrow wounds or lacerations. Additional staff members were able to return by January 6. During this time one staff member required escort through the town to assure his safety and some staff had to quarter at a nearby police station to avoid travel through hostile areas of town.

#### Patient visits

**Figure 4:**
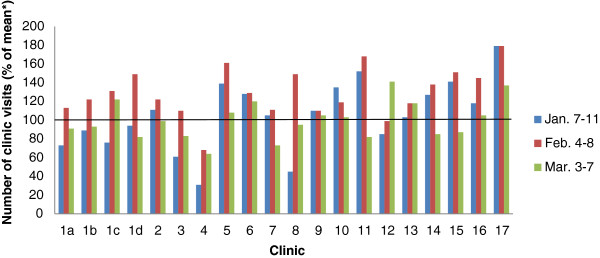
**Number of patient visits in AMPATH clinics (January –March 2008) following the election crisis.** *The number of patients visiting each clinic in the first week of each month (January-March) following the election crisis was calculated as the percentage of the mean number of weekly patient visits during the 6 months prior to the post-election crisis.

The majority of clinics (12/20) had more clinic visits during their first week open than their mean weekly clinic visits over the previous six months, leading to an overall patient volume within AMPATH of 105% (Figure 4). However, of the seven clinics located in the areas most directly affected by the violence, six had lower than anticipated patient volumes during the first week of opening. This trend continued over the subsequent two weeks with all seven clinics experiencing a decreased number of visits at week 2, and five of the seven clinics showing decreased volume at week 3.

Trends in patients’ visits at all AMPATH clinics are shown in Table 1. The number of previously scheduled patient appointments was decreased during the period of the holidays and elections due to the anticipated travel of patients to their rural homes. In January 2008 the number of missed visits rose to 40.7%, up from an average 23.1% (21.9-23.9) in the preceding three months. However, the number of unscheduled visits in January increased to 42.4%, a significant increase from the average of 27.2% (26.8-27.7) in the period October to December. The number of missed patient visits declined during February and March while the number of unscheduled visits remained high (36-42%), suggesting patients who had previously missed visits returned to clinic during this time.

**Table 1:** Trends in patient attendance over the period across AMPATH sites and at Burnt Forest clinic

**All AMPATH clinics**	**OCT ′07**	**NOV ′07**	**DEC ′07**	**JAN ′08**	**FEB ′08**	**MAR ′08**
**Total scheduled appointments**	33613	28183	27165	23259	21598	25891
**Total “Missed” Visits (a)**	7358	6749	6351	9463	7253	6851
**Total “On-time” visits (b)**	26255	21434	20814	13796	14345	19040
**Total “Unscheduled” visits (c)**	9616	8212	7756	10153	10378	10834
**Total “Turnover” (Scheduled + Unscheduled visits) (d)**	35871	29646	28570	23949	24723	29874
**Burnt Forest clinic**	**OCT ′07**	**NOV ′07**	**DEC ′07**	**JAN ′08**	**FEB ′08**	**MAR ′08**
**Total scheduled appointments**	1211	1124	960	644	470	517
**Total “Missed” Visits (a)**	329	372	327	527	180	285
**Total “On-time” visits (b)**	882	752	633	117	290	232
**Total “Unscheduled” visits (c)**	454	367	361	307	381	453
**Total “Turnover” (Scheduled + Unscheduled visits) (d)**	1336	1119	994	427	671	685

KEY:

(a) Those who did not attend on the ‘exact’ appointment date.

(b) Those that attended on the exact appointment date.

(c) Those that attended on dates other than their exact appt. dates (either before or after the scheduled date).

(d) The total number of patients who attended clinic.

#### Burnt Forest patient visits

Burnt Forest reported one CO and one nurse saw 85 patients in the first week they were open. The number of patients presenting to clinic increased in the second week (222 patients seen by two COs and one nurse), but decreased again in the following weeks as this area remained largely unstable due to continued tribal separation and sporadic outbursts of violence (Table 2). During the initial weeks of clinic operations, visits were generally unscheduled and very short as patients and staff feared for their safety. Many patients reported the loss of their antiretrovirals due to fire or rapid evacuation. Patients experience difficulty in safely moving through town to access the clinic. As such, the clinic contacted patients with mobile phones and asked them to contact other patients in their area to let them know that they could access care from AMPATH at a satellite clinic (30 km north) or in Eldoret (40 km northwest). Attendance trends for Burnt Forest (Table 1) showed a significant increase in missed visits in January to 81.8%, up from an average 31.5% (27.2-434.1) in the preceding three months. However, as was the trend in all AMPATH clinics, the number of unscheduled patient visits in January rose to 71.9% from an average 34.4% in October through December 2007.

**Table 2:** Patient return to Burnt Forest Clinic

**Mean number of weekly patient visits in 6 months preceding the crisis**	**Number of documented clinic visits January 7–11, 2008 (% of mean)**	**Number of documented clinic visits January 14–18, 2008 (% of mean)**	**Number of documented clinic visits January 21–25, 2008 (% of mean)**	**Number of documented clinic visits February 4–8, 2008 (% of mean)**	**Number of documented clinic visits March 3–7, 2008 (% of mean)**
274	31	81	44	68	64

#### ART Availability

All clinics reported having adult and pediatric antiretrovirals available at the time of their reopening. During the week of January 14–18, two clinics located in areas directly affected by the violence could not provide a full month’s supply of antiretrovirals, the amount of ART usually provided to patients. Both clinics had fully restocked their medications by January 21. All patients receiving incomplete supplies of medications were asked to return the following week in order to receive additional antiretrovirals. It is noted that during this time both AMPATH and non-AMPATH patients could seek ART refills from any AMPATH clinic by showing their pills bottles or knowing their current regimen.

#### Impact on documentation

In the weeks following the crisis 7/19 clinics reporting data on their post-election functioning (all excepting Mosoriot) experienced disruptions in the normal collection of patient data due to the need for rapid and streamlined visits to maintain patient safety and to accommodate decreased staff numbers. All clinics had been instructed at minimum to keep a paper log of patient visits. Two clinics (including Burnt Forest) reported that the only data collection during the first week of their opening was the maintenance of a list of patients seen including their ID numbers. Those two clinics as well as five others required one to four weeks to fully normalize their data collection. Due to safety issues on the roads movement of paper forms to the data management center in Eldoret was temporarily halted.

#### Tracking missing patients

Although some clinics were able to function normally throughout the crisis, others had a significant number of patients who missed appointments and needed to be located (Figure 4). Using the AMRS, clinics were able to generate a list of patients receiving ART and those patients who had missed previously scheduled appointments. The Outreach Department, with the support of the mobile clinic and outreach arm, utilized the resulting list to contact patients via phone or face to face in homes or IDP camps.

#### Burnt Forest patient tracking

Through the use of phone calls, patient tracking and review of patient logs from other sites, Burnt Forest clinic was able to confirm that 494/983 (50%) of their patients on ART pre-violence had either received care from another program (3), an AMPATH clinic (369) or an IDP camp (122) by February 18, 2008. In order to locate those patients identified as missing, 15 local Community Healthcare Workers (CHWs) were recruited and asked to identify and encourage patients in the community to return to clinic. When a group of patients who did not feel safe traveling to the clinic was identified within a catchment area, the CHW arranged for a mobile clinic team to come to the area and provide antiretrovirals. In addition, several patients in communities served by the Burnt Forest clinic became resource persons for care for patients who were not located in IDP camps. These individuals would travel to the clinic and carry back supplies of medications to other patients in their communities, or they would arrange for local patients to congregate at their home at pre-arranged times so that Burnt Forest clinic personnel could deliver medications to many patients at once within the community.

### IDP Camp outreach teams

#### Team structure

Twenty-three IDP camps fell within or near the catchment area of the AMPATH clinics. On January 7, 2008 teams consisting of a staff member from administration, nursing, community mobilization and psychosocial support made the first visits to the IDP camps. Based on their observations they assembled response teams composed of nurses, outreach workers, psychosocial support staff, nutritionists and social workers that were sent to the camps to provide the care and support needed. Mobile teams were each assigned to several camps and rotated their visits between camps on a weekly basis. These teams provided ART, TB treatment, opportunistic infection (OI) prophylaxis and other support services. A Medical Officer or Clinical Officer made weekly visits to the camps to provide more advanced medical care and consultation. In total, teams saw 1,420 HIV infected individuals in the IDP camps. Of those, 1,290 were AMPATH patients and 637 were on ART. Of the 1,420 patients seen, 738 had data captured by the “Field Encounter Form– Internally Displaced AMPATH Patients” which was implemented after the initial rapid response. Of these, 76% were AMPATH patients, 85% were adults and 77% were female. Two hundred ninety-one (39%) reported their home had been destroyed and 137 (19%) reported some interruption of their ART.

#### Identification of AMPATH patients

Within the IDP camps, in order to gain patient recognition, staff initially wore shirts that designated them as AMPATH personnel. This practice was quickly abandoned as staff realized patients were reluctant to approach them due to the fear of identifying themselves as HIV positive by their association with the AMPATH staff. Subsequently, the teams used word of mouth through peer educators affiliated with AMPATH and the patients themselves to spread the news that the team had arrived in the camp.

#### Distribution of supplies, food and money

In addition to ART, food was distributed to over 1,290 AMPATH patients and their dependents residing in the IDP camps through AMPATH’s food distribution program. Minimal supplies such as plastic tarps (250), blankets (500), cooking utensils (335), sanitary napkins (681) and underwear (181) were also provided. Whenever possible, the food and supplies were provided to patients at a clinic in order to reduce patient stigma, conflicts with non-AMPATH IDP camp residents and interference with the missions of other relief agencies. Small amounts of money were available to patients to facilitate their transport to the clinics to collect supplies or occasionally to directly purchase supplies. These funds were made available from the Recovery Fund, an account established in January 2008 to receive donations from individuals, foundations, churches and other entities.

#### Psychosocial support

Most initial outreach teams had a psychosocial support staff member. They developed patient support groups in the camps serving upward of 70 patients at one time. Individual counseling needs were also addressed with 632 patients receiving over 4,000 counseling sessions in the IDP camps in the region. Special attention was given to the counselors themselves, with support sessions conducted by a mental health specialist and a counseling supervisor. Emphasis was placed on addressing the individual needs of the counselors and providing them with increased awareness of their own issues so that they could deal with them appropriately and prevent future burnout.

#### Burnt Forest and the Nakuru IDP camp

Within 3 weeks of the crisis, Burnt Forest staff was providing support to patients at 13 different IDP camps and satellite sites both within the Burnt Forest catchment and in areas east and southeast of Burnt Forest health center. One hundred-twenty kilometers southeast of the Burnt Forest clinic, outside of AMPATH’s catchment area, one of the largest IDP camps was established in Nakuru, Kenya. This camp supported 14,000 individuals [25], including over 100 AMPATH patients. Due to the large number of AMPATH patients within the Nakuru IDP camp, its distance from other AMPATH clinics and unsafe travel conditions, a nurse and an outreach worker from Burnt Forest, who were also displaced, were assigned to the camp full time until September 2008 when all patients were relocated. The two staff members stationed in this camp were from the Burnt Forest clinic and had been displaced and were living in the Nakuru IDP camp. While some patients in this camp obtained their ARV supplies from the Rift Valley Provincial General Hospital, Nakuru, the majority received ART and other logistical support from the AMPATH center in Eldoret. A physician and CO from Burnt Forest clinic visited the camp every two weeks to deliver medicines and supplies and see patients requiring medical consultation.

### Staff assistance

A number of AMPATH clinic and ancillary staff were directly affected by the post-election violence. In some cases their homes were burned, property was destroyed or stolen or they were forced to leave their communities. These staff members were assisted by other staff as well as through donations given for humanitarian expenditures. When possible staff members opened their homes to displaced staff members and their families until other housing could be secured. Those staff members identified as being at the highest risk were assisted in evacuation, in some cases with security escorts and relocation allowances. Monetary support to assist with housing, food or transportation could be requested in writing through the Recovery Fund. Provisions of food and cell phone airtime were also provided through this fund. Psychological support was provided to staff, as counselors met with individuals to discuss issues of fear, stress, loss or grief. Formal psychosocial education sessions were held in late January 2008 for approximately 100 staff members to discuss the recent traumatic events, discuss staff needs and to provide coping strategies to deal with the trauma and ongoing tensions resulting from the violence. Debriefing sessions were held by counselors in 14 departments at the Eldoret AMPATH center and at the clinics in Burnt Forest and Turbo to address fears and distrust among co-workers stemming from the ethnic focus of the post-election violence. In total 300 staff members received counseling.

## Discussion

The instability following the 2007 Kenyan presidential election is an example of a countrywide disruption that affected all aspects of life including housing, transportation, education, commerce and healthcare. In this paper, we have shown that the AMPATH system maintained a high level of functioning with minimal gaps in service delivery. Prompt implementation of a response system allowed most patients’ uninterrupted access to ART or rapid return to care. We identify lessons learned based on the experience with the AMPATH response and recognize system factors that facilitated the success of that response. We believe that the lessons learned during the Kenyan post-election crisis may be valuable to other ART programs located in regions of the world prone to instability due to political or natural crises.

### Lesson 1: A rapid organizational response is critical

Minimizing disruptions to healthcare systems during times of crisis requires forethought, planning and organization. In analyzing AMPATH’s response to the crisis a key factor in the success of the response was the organization’s ability to rapidly establish a multidisciplinary response team. As such, one recommendation arising from our experience is that a Crisis Response Team should be established in advance as part of the structure of large HIV care and treatment programs located in potentially unstable regions. Médecins Sans Frontières (MSF) similarly designates staff to prepare for instability [21] and create an Emergency Preparedness Plan [19]. The Response Team should include representatives from program leadership as well as key departments (i.e. pharmacy, social work, psychosocial, nutrition and outreach). Members should be aware of their designated roles and responsibilities and alternative representation should also be designated. Priorities for patient care should be clearly defined in advance and strategies for restructuring care delivery to compensate for staff shortages, increased patient load, potential supply disruptions and curtailed record keeping should be addressed.

At the time of a crisis, this team should be immediately assembled (in person, electronically, or via phone) and tasked with the rapid assessment of the situation to determine the impact of the crisis on the safety and stability of the communities surrounding the healthcare system, the structural and functional integrity of the clinic(s) and the extent to which displacement has affected staff and patients. These assessments should be used to tailor the team’s response to the crisis, which might include the use of multi-disciplinary field teams to address patient needs, public service announcements (address the identified needs of the specific population) [19-21,26], activation of an information hotline (utilizing numbers from each major phone carrier in order to minimize problems with patient accessibility [19]), activation of community networks, and establishment of alternative care delivery sites [21]. The developed response mechanism, including key personnel and procedures, should be reviewed and evaluated on a regular basis.

### Lesson 2: Clinic autonomy and self-sufficiency is indispensable

The ability of the AMPATH clinics to function autonomously in the early period of the crisis was key to the success of the systems response because of disruptions in transport, communications, and issues related to safety. This finding supports the recommendation that within a multi-clinic system the capacity for some autonomous operations, for at least limited time periods, should be developed in the individual clinics. This would include ensuring the staff members are cross trained to take additional or alternative roles [19-21], maintenance of at least one month stock of supplies and medications, and ensuring that emergency funds are available at the clinic to meet unforeseen operating costs. The optimal quantity of medications and supplies to be stocked requires local assessment of patient volume, storage capacity, anticipated duration of disruption(s), and the program’s capacity for alternative means for drug/supply delivery. MSF has made similar recommendations for maintaining increased drug supplies at clinics and distributing extended supplies of patient medications when instability is anticipated as was done by AMPATH practitioners, particularly in Burnt Forest, at the time of the 2007 Kenyan elections and holidays [19,20]. In times of unanticipated crisis, MSF had recommended the rapid distribution of pre-prepared “runaway stock” (1–3 months of ART and OI prophylaxis) to patients [17]. In addition, clinics should be given license to adjust patient flow and documentation in order to accommodate reduced staff numbers, and in some cases, increased patient numbers. Streamlining of patient visits and the minimization of paperwork as used by both AMPATH and MSF can [19] prevent disruptions in patients’ ART at the reasonable expense of limited data collection.

### Lesson3: Patient knowledge of their HIV status and treatment regimen is necessary

Promoting patient self-sufficiency in seeking care is also important. Patients should receive ongoing education to ensure that they are familiar with the status of their HIV disease (CD4 count, viral load, history of OIs) and know the component drugs of their ART regimen. MSF has utilized patient “passports” that include previous and current ART regimens and any known side effects [19]. AMPATH currently prints a “Clinical Summary” for health providers at each patient visit that shows the patient’s current ART regimen and OI medications, current and past problem list and a chronological listing of the patients weights, hemoglobin counts, CD4 counts, viral loads and SGPTs. As this information is already available, it could be provided to patients at each scheduled visit with instructions to keep it in a safe place such that the patient could present it to other providers if needed in the future.

### Lesson 4: Patient and community networks are essential

At Burnt Forest the strong ties between the community, including both patients and other community members, and the clinic was found to be invaluable to ensuring that patients received uninterrupted supplies of ART. The recommendation based on this finding is that clinics/ART treatment programs develop significant relationships with their surrounding communities. This could include establishing patient contact lists and facilitating self-forming patient groups to aid communication between the clinic and the community of patients both for the enhancement of routine care and to facilitate rapid communication during times of disruption. Clinics/programs utilizing CHWs or expert patients will want to consider how to incorporate these key members of the community into their crisis response plan. As emphasized previously by MSF, each clinic/program must project an image of neutrality in its healthcare delivery in order to maintain the support of the community [21], especially when there is discord among community members as was seen in Burnt Forest.

### Lesson 5: Ensuring staff security and access to basic needs is fundamental

The Kenyan post election violence impacted the full cadre of staff working in the AMPATH clinics. Staff members experienced displacement, food insecurity, transportation issues, and security concerns. By addressing these issues AMPATH facilitated the return of staff to functional positions within the system. For individuals in targeted ethnic groups in high risk areas, that included relocation to positions outside their primary clinic. For others it required finding alternative housing, providing funds to replace damaged or stolen property, or to buy food. This finding supports the recommendation that programs make arrangements for rapidly assessing staff needs and then provide with a plan to meet those needs either internally or by establishing agreements with organizations whose missions can provide for those needs.

### Lesson 6: The ability to rapidly identify and track all patients requiring ART is key

At Burnt Forest, in particular, many patients were unable to access the clinic due to security issues and/or displacement. The AMPATH electronic medical record (EMR) system was a critical tool in identifying patients who had not returned for their scheduled appointments and providing a list of patients that were at risk for ART disruptions. This finding leads to the recommendation that programs should have the capacity to rapidly generate lists of patients who have not returned for care within a specified period of time. In the case of ART care systems/clinics with EMRs this means that the programming to generate such lists should be developed prior to a potential crisis and be ready to be utilized when needed. In the case of programs without EMRs, paper based systems for identifying patients at risk for ART disruption should be developed and tested. However, generation of such lists is only useful if the program maintains up to date contact information on all patients. Contact information should include cell phone number(s) if available as well as the patient’s complete address. In countries where street addresses are uncommon a detailed description of how to get to a patient’s house including a map should be maintained in the patient’s file. Decentralization of EMR capacity to local clinic sites would improve the ability for individual clinics to track patients, verify treatment regimens, and monitor treatment locally. AMPATH is currently working to decentralize EMR operations to all clinics.

Some clinic systems have designated staff (outreach workers, trackers, etc.) and systems for locating patients who have not returned to clinic within a specified time frame. These individuals should be included in a crisis response team and provide insight into the development of plans to contact patients at times of social disruptions. Clinics without this capacity should identify and train staff and develop systems for providing outreach to patients who are at risk of ART disruption. This may include the use of patient networks as described above.

### Study limitations

Although the majority of patients were able to return to care in a timely manner, this case study is limited in its ability to quantify the actual proportion of patients that had interruptions in care. In order to determine this number, all patients actively receiving ART prior to the elections would need to be known and then compared against a list of all patients returning to care within a specified amount of time following the elections. However, patients’ scheduled return dates were not consistently recorded in their charts and may have varied during this time due to the holidays and upcoming elections. Patients may have returned to clinic before or after their scheduled date with an unknown interruption in ART. Furthermore, due to the disruptions in some clinics, only pharmacy logs were kept to record ART distribution with no formal encounter form filled to log a patient’s visit. Furthermore, preparations for the holidays and elections affected the average number of days that drugs were given for patients at the time of the election. Drug refills for patients normally attending the most affected clinics is difficult to determine as patients were able to receive refills at other clinic sites, ancillary clinics, non-AMPATH clinics and IDP camps.

In the two months following the election specific documentation of the location and status of 50% of the patients on ART at Burnt Forest was recorded. Individual patient tracking was then discontinued and specific patient level data is not available to allow for a true assessment of how many of the remaining patients were lost to follow-up due to the crisis. An analysis of aggregate data from AMRS of the patient cohort starting ART prior to the crisis was done. It showed that during the two years immediately following the crisis the rate of patients lost to follow-up at Burnt Forest was greater than AMPATH in its entirety (where the majority of patients were not affected by the crisis) by 3-4% but equalized by 2010.

This case report highlights the response of the AMPATH HIV clinic system to the crisis following the 2007 Kenyan elections. We acknowledge that the experiences and responses documented here may not be applicable to all settings or crisis situations.

## Conclusion

This case study highlights the challenges in delivering healthcare, specifically ART, during a time of social disruption. This case illustrates the importance of advance planning to develop programs that can function during a crisis and emphasizes the need for a rapid programmatic response, ability of clinics to function autonomously, ensuring patients are knowledgeable about their HIV status and treatment, use of community and patient networks, addressing staff needs and developing effective patient tracking systems.

## Abbreviations

AMPATH: USAID-academic model providing access to healthcare; ART: Antiretroviral therapy; HIV: Human immunodeficiency virus; AMRS: AMPATH medical record system.

## Competing interests

The authors declare they have no competing interests.

## Authors’ contributions

SPG conducted key informant interviews, did qualitative and quantitative analysis and drafted the manuscript. SN helped conceive the case study, contributed to the acquisition of data, supervised the qualitative and quantitative analysis, provided extensive critical revision to the manuscript and gave final approval for publication. SK made contribution to the acquisition of data and provided critical revision of the manuscript. HS and JW made substantial contributions to the acquisition of data and provided critical revision of the manuscript. PB contributed to the conception and design of the study, contributed to the acquisition of data and provided critical revision of the manuscript and gave final approval for publication. JES made substantial contribution to the acquisition of data, supervised the qualitative and quantitative analysis, provided extensive critical revision to the manuscript and gave final approval for publication. JS helped conceive the case study, contributed to the acquisition of data and provided critical revision of the manuscript. RO made substantial contributions to the acquisition of data and provided critical revision of the manuscript. CC, CG, FK and CO made substantial contributions to the acquisition of data and provided critical revision of the manuscript. ES contributed to the analysis and interpretation of data. AS made contribution to the acquisition of data and provided critical revision of the manuscript. KWK helped conceive the case study, supervised the qualitative and quantitative analysis, provided extensive critical revision to the manuscript and gave final approval for publication. All authors read and approved the final manuscript.

## Authors’ information

SPG is an Assistant Research Scientist at IUSM in the Division of Infectious Diseases. SN is the Associate Program Manager for AMPATH. SK is the AMPATH Program Manager. HS is the Clinical Officer In-Charge at the AMPATH Burnt Forest clinic. JW is a Research Consultant with the OSCAR foundation and WALTHER project within AMPATH. PB is an Associate Research Professor of Medicine at IUSM, Co-Field Director of Research for AMPATH, oversees monitoring and evaluation for AMPATH and is a Visiting Lecturer at the Moi University School of Medicine. JES is an Associate Professor of Medicine at IUSM, an Affiliated Scientist at Regenstrief Institute, a Co-Field Director of Research for AMPATH and a Guest Lecturer at Moi University College of Medicine. JS is an Assistant Lecturer in Moi University School of Public Health and a consultant with AMPATH Primary Health Care. RO is a registered Community Health Nurse with a background in sociology who is the current TB/HIV Liaison Officer in AMPATH. CC is the Social Work Manager for AMPATH in Kenya. CG was the AMPATH Food Distribution Manager at the time of the crisis and is currently a PhD candidate at Griffith University in the School of Public Health. FK is the senior Nutritionist at Moi Teaching and Referral Hospital and the former Nutrition Services Manager for AMPATH. CO is Assistant Program Manager for Psychological Counseling at AMPATH Plus. ES is a Data Manager for AMPATH in Kenya. AS is Associate Program Manager for AMPATH Primary Healthcare. KWK is Director of the Division of Infectious Diseases and an Associate Professor of Medicine at IUSM and Co-Field Director of Research Emerita for AMPATH.
